# The Simultaneous Presence of Isolated Tumour Cells and Bone Marrow Micrometastases in Stage I and II Colon Cancer—Challenging the Theory of a Chronological Pathway of Tumour Cell Dissemination

**DOI:** 10.1007/s00268-021-06399-4

**Published:** 2021-12-27

**Authors:** Michaela Ramser, Rene Warschkow, Carsten T. Viehl, Christoph Kettelhack, Andreas Zettl, Leonard A. Lobbes, Markus Zuber, Benjamin Weixler

**Affiliations:** 1 Clarunis University Center for Gastrointestinal and Liver Diseases, St. Clara Hospital and University Hospital Basel, Basel, Switzerland; 2 Department of Surgery, Cantonal Hospital Olten, Olten, Switzerland; 3 Department of Surgery, Cantonal Hospital St. Gallen, St. Gallen, Switzerland; 4 Institute of Medical Biometry and Informatics, University of Heidelberg, Heidelberg, Germany; 5 Department of Surgery, Hospital Center Biel/Bienne, Biel/Bienne, Switzerland; 6 Histopathology & Cytology, Viollier AG, Basel, Switzerland; 7 Department of General, Visceral, and Vascular Surgery, Charité University Hospital, Campus Benjamin Franklin, Berlin, Germany

## Abstract

**Background:**

According to the common tenet, tumour progression is a chronological process starting with lymphatic invasion. In this respect, the meaning of bone marrow micrometastases (BMM) in patients with lymph node negative colon cancer (CC) is unclear. This study examines the relationship of isolated tumour cells (ITC) in sentinel lymph nodes (SLN) and BMM in patients in early CC.

**Methods:**

BM aspirates were taken from both pelvic crests and in vivo SLN mapping was done during open oncologic colon resection in patients with stage I and II CC. Stainings were performed with the pancytokeratin markers A45-B/B3 and AE1/AE3 as well as H&E. The correlation between the occurrence of ITC+ and BMM+ and their effects on survival was examined using Cox regression analysis.

**Results:**

In a total of 78 patients with stage I and II CC, 11 patients (14%) were ITC+, 29 patients (37%) BMM+. Of these patients, only two demonstrated simultaneous ITC+ /BMM+. The occurrence of BMM+ was neither associated with ITC+ in standard correlation (kappa = − 0.13 [95% confidence interval [CI] = − 0.4–0.14], *p* = 0.342) nor univariate (odds ratio [OR] = 0.39, 95%CI:0.07–1.50, *p* = 0.180) or multivariate (OR = 0.58, 95%CI: 0.09–2.95, *p* = 0.519) analyses. Combined detection of ITC+ /BMM+ demonstrated the poorest overall (HR = 61.60, 95%CI:17.69–214.52, *p* = 0.032) and recurrence free survival (HR = 61.60, 95%CI: 17.69–214.5, *p* = 0.032).

**Conclusions:**

These results indicate that simultaneous and not interdependent presence of very early lymphatic and haematologic tumour spread may be considered as a relevant prognostic risk factor for patients with stage I and II CC, thereby suggesting the possible need to reconsider the common assumptions on tumour spread proposed by the prevalent theory of sequential tumour progression.

## Introduction

Prognosis of patients with colon cancer is still limited despite improved surgical techniques, guidelines to assure an adequate lymph node yield and multimodal oncological therapy. Patients with node-negative disease (i.e. Union for International Cancer Control (UICC) stage I and II) and absence of risk factors (T4, perforation, bowel obstruction, < 12 LN analysed, poor histologic grade, peritumoral lymphovascular invasion (LVI)) are considered tumour-free after adequate surgery without indication for adjuvant therapy [1, 2]. Nevertheless, a significant number of patients still shows a clearly impaired survival [3, 4], thus suggesting occult tumour dissemination already at the initial histological staging.

According to the prevailing understanding of metastasis development as a sequential progression, tumour cells first spread to local lymph nodes (LN) where they form metastatic deposits and then eventually disseminate to higher tier LN and finally become blood borne with the formation of distant metastasis [5]. Histological staging identifies the earliest LN deposits as either isolated tumour cells (ITC) or micrometastasis [6]. In colon cancer, nodal micrometastases are thereby defined as tumour deposits of 0.2 mm to ≤ 2 mm and are classified as nodal positivity, upstaging tumours to stage III (since the 6th edition of the UICC: pN1(mi)) [7–9]. LN with ITC on the other hand are harbouring either single tumour cells or clusters of tumour cells of ≤ 0.2 mm and are considered as negative LN (pN0(i +)) [10]. Despite their small size, ITC have been reported to impact survival in early stage colon cancer [11, 12]. These “occult” metastases are difficult to find with standard histopathological techniques, and it has been demonstrated that sentinel lymph node (SLN) mapping with multilevel sectioning and immunohistochemical staining improves their detection rates [6, 13]. SLN have previously been shown to represent the first draining LN in the hierarchical lymphatic draining system from colon cancer and harbour metastases significantly more often than all other LN in a resected specimen [6, 14–17]. A more in depth-analysis of the SLN is therefore considered the best way to avoid understaging [18].

Interestingly, a considerable proportion of node negative patients nevertheless are diagnosed with metachronous distant metastases, challenging the hypothesis of sequential progression [19, 20]. In fact, data suggest that a synchronous lymphatic and haematologic spread might be an alternative hypothesis that would put into question much of the current tumour understanding and treatment strategies [21, 22]. It has been suggested that LN metastases serve only as an indicator of increased likelihood of metastasis, but that they are not themselves contributing to the seeding in the sense of a metastatic cascade [5, 23–26]. In fact, a review on the role of lymphadenectomy in a diversity of solid tumours concluded that lymphadenectomy does not improve overall survival but should be merely conceived as a tool of staging, regional control and as a prognostic indicator [27].

The role of circulating tumour cells in the peripheral blood and disseminated tumour cells, e.g. to the bone marrow (BM), are both considered early manifestations of subsequent overt metastasis and have been researched for many years and in a variety of tumours [28–31]. Nevertheless, the significance of such tumour cells in the BM in patients with node-negative colon cancer is not known and the very early cascade of tumour cell spread to LN and the BM remains unclear.

With this study, we wanted to investigate the prognostic relevance of early tumour cell presence in the first draining LN and tumour cells in the BM in patients with non-metastatic and node-negative colon cancer and the respective interdependent relationship in order to evaluate their respective prognostic impact, thereby analysing suggested evidence regarding early sequential or simultaneous tumour progression.

## Patients and methods

### Study settings

**Fig. 1:**
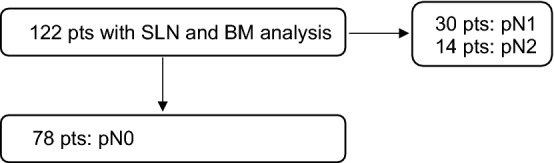
Consort diagram

The here included patients represent a subgroup analysis of a prospective multicenter study (NCT00826579). More precisely, it is an analysis of the node-negative patients [32]. Node positive patients were excluded to obtain a homogenous group of patients with truly early colon cancer. Studying isolated tumour cells in patients with nodal macrometastases in other lymph nodes possibly would have influenced the results to an unpredictable degree. Nodal micrometastases defined as tumour deposits of 0.2 mm to ≤ 2 mm were thereby considered as positive lymph nodes and accordingly upstaging to stage III (since the 6th edition of the UICC: pN1(mi)) and not considered for this analysis [7–9]. A consort diagram is shown in Fig. 1. This study was performed at three academic and university-affiliated hospitals in Switzerland and patients were included from 05/2000 until 12/2006. The mean follow-up period was 6.5 years (IQR 5.3–8.3).

The study protocol was approved by the ethical committees of all participating centres. The inclusion and exclusion criteria for the study population have already been reported [30]. In summary, patients with preoperatively verified colon cancer were eligible for the study. Exclusion criteria were defined as stage IV disease, rectal cancer, history of other solid malignancies, and previous abdominal cancer surgery. Written informed consent was obtained prior to surgery. For the present study, only patients with stage I and II colon cancer were considered. Tumours were staged according to the 6th version of the tumour-node-metastasis (TNM) classification system [7]. Tumours with LN micrometastases (pN1(mi)) were therefore considered as stage III and were not considered for this analysis.

All patients received an open oncologic colon cancer resection. The technical details of SLN mapping and BM aspiration have been reported previously [33, 34]. In short, after induction of general anaesthesia and before the oncologic resection, BM aspirates were taken from both pelvic crests. [30, 34]. Detection of ≥ 1 tumour cells was considered as BMM+.

For the SLN mapping, isosulfan blue was injected in vivo into the subserosa around the tumour. All LN that coloured blue within 10 min were marked as SLN [6, 33]. Five serial sections were then obtained at 3 different levels of each SLN. These were stained with H&E and if H&E was negative, additional immunostainings with the pancytokeratin marker AE1/AE3 (DakoCytomation, Glostrup, Denmark) were performed.

Adjuvant chemotherapy was recommended for stage II patients featuring risk factors (i.e. < 12 LN analysed, T4 tumour, LVI, poor differentiation or perforation) [2]. ITC in the LN (and BMM+) were not deemed an indication for adjuvant chemotherapy. Postoperative surveillance was conducted following national surveillance guidelines [35, 36].

### Statistical analyses

Analyses were done by the R statistical software (www.r-project.org). A two-sided *p*-value < 0.05 was considered statistically significant. Chi-Square statistics were used to analyse proportions and ANOVA tests to analyse continuous variables. The interrelationship of ITC+ and BMM+ was evaluated using Kappa statistics with the 95% confidence intervals [37]. Further, univariable and multivariable logistic regression analyses were conducted to analyse the predictive value of ITC+ for BMM+ and of BMM+ for ITC+. Due to complete and quasi-complete separation (occurrence empty categories), Firth’s correction to the likelihood (penalized maximum likelihood) was used [38, 39]. For logistic regression, *p*-values were computed by likelihood-ratio-tests and Wald-type confidence intervals were calculated. The impact of ITC+ and BMM+ as prognostic factors for overall and disease-specific survival was evaluated in univariable and multivariable Cox regression analyses. For Cox regression, *p*-values were calculated by likelihood-ratio-tests and Wald-type confidence intervals were estimated.

## Results

In a total of 78 patients with stage I or II colon cancer, in vivo SLN mapping and BM aspiration were successfully performed. Patient demographics and histopathologic tumour data are shown in Table 1.

**Table 1:** Baseline characteristics of all patients included in the analysis (*n* = 78)

	Total	ITC− /BMM−	ITC+ /BMM−	ITC− /BMM+	ITC+ /BMM+	
	*n* = 78	*n* = 40	*n* = 9	*n* = 27	*n* = 2	
UICC stage						0.179 A)
I	22 (28.2%)	13 (32.5%)	0	9 (33.3%)	0	
II	56 (71.8%)	27 (67.5%)	9 (100%)	18 (66.7%)	2 (100%)	
Tumour stage, *n* (%)						0.547 A)
pT1	7 (9.0%)	4 (10.0%)	0	3 (11.1%)	0	
pT2	15 (19.2%)	9 (22.5%)	0	6 (22.2%)	0	
pT3	46 (59.0%)	23 (57.5%)	6 (66.7%)	15 (55.6%)	2 (100%)	
pT4	10 (12.8%)	4 (10.0%)	3 (33.3%)	3 (11.1%)	0	
Number of LN						0.591 B)
Median (IQR)	23.5 (19.0–31.0)	23.0 (18.8–31.0)	25.0 (22.0–27.0)	25.0 (19.5–31.5)	19.5 (19.2–19.8)	
Range	7.0–62.0	14.0–56.0	15.0–62.0	7.0–57.0	19.0–20.0	
Validated, *n* (%)	78 (100%)	40 (100%)	9 (100%)	27 (100%)	2 (100%)	
Number of SLN						0.012 B)
Median (IQR)	3.0 (2.0–4.0)	2.0 (1.8–4.2)	6.0 (5.0–7.0)	3.0 (1.5–3.0)	3.0 (2.5–3.5)	
Range	0.0–14.0	1.0–11.0	1.0–14.0	0.0–7.0	2.0–4.0	
Validated, *n* (%)	78 (100%)	40 (100%)	9 (100%)	27 (100%)	2 (100%)	
SLN with ITC (IHC)						< 0.001 B)
Median (IQR)	0.0 (0.0–0.0)	–	1.0 (1.0–2.0)	–	2.0 (1.5–2.5)	
Range	0.0–4.0	–	1.0–4.0	–	1.0–3.0	
Validated, *n* (%)	78 (100%)	40 (100%)	9 (100%)	27 (100%)	2 (100%)	
Number of positive cells in BM						< 0.001 B)
Median (IQR)	0.0 (0.0–1.8)	–	–	2.0 (1.0–5.0)	5.5 (4.2–6.8)	
Range	0.0–95.0	–	–	1.0–95.0	3.0–8.0	
Validated, *n* (%)	78 (100%)	40 (100%)	9 (100%)	27 (100%)	2 (100%)	
Lymphovascular invasion, n (%)						0.022 A)
Absent	70 (89.7%)	37 (92.5%)	6 (66.7%)	26 (96.3%)	1 (50.0%)	
Present	8 (10.3%)	3 (7.5%)	3 (33.3%)	1 (3.7%)	1 (50.0%)	
Grade, *n* (%)						0.692 A)
G2	55 (70.5%)	30 (75.0%)	7 (77.8%)	17 (63.0%)	1 (50.0%)	
G3	23 (29.5%)	10 (25.0%)	2 (22.2%)	10 (37.0%)	1 (50.0%)	
Tumour site, n (%)						0.104 A)
Right colon	35 (44.9%)	14 (35.0%)	2 (22.2%)	18 (66.7%)	1 (50.0%)	
Transverse Colon	12 (15.4%)	7 (17.5%)	3 (33.3%)	2 (7.4%)	0	
Left colon	31 (39.7%)	19 (47.5%)	4 (44.4%)	7 (25.9%)	1 (50.0%)	
Chemo therapy, n (%)						0.284 A)
No	66 (84.6%)	36 (90.0%)	6 (66.7%)	22 (81.5%)	2 (100%)	
Yes	12 (15.4%)	4 (10.0%)	3 (33.3%)	5 (18.5%)	0	
CEA preoperative						0.888 B)
Median (IQR)	1.8 (0.8–3.4)	1.8 (0.7–2.5)	1.6 (1.1–3.8)	1.7 (0.8–3.3)	3.0 (2.1–3.8)	
Range	0.0–41.0	0.0–41.0	0.6–38.0	0.0–15.0	1.2–4.7	
Validated, *n* (%)	70 (89.7%)	33 (82.5%)	9 (100%)	26 (96.3%)	2 (100%)	
CEA postoperative						0.738 B)
Median (IQR)	1.1 (0.8–1.8)	1.3 (0.8–1.8)	1.1 (0.5–1.6)	1.0 (0.9–2.0)	0.7 (0.7–0.7)	
Range	0.5–12.9	0.5–12.9	0.5–3.2	0.5–4.8	0.7–0.7	
Validated, *n* (%)	39 (50.0%)	17 (42.5%)	5 (55.6%)	16 (59.3%)	1 (50.0%)	
Sex, *n* (%)						0.464 A)
Male	30 (48.7%)	16 (40.0%)	6 (66.7%)	15 (55.6%)	1 (50.0%)	
Female	40 (51.3%)	24 (60.0%)	3 (33.3%)	12 (44.4%)	1 (50.0%)	
Age						0.692 B)
Median (IQR)	74.5 (66.6–78.6)	75.8 (68.0–78.2)	73.6 (65.1–74.9)	71.1 (60.4–82.8)	77.4 (77.1–77.7)	
Range	27.3–92.2	27.3–87.5	55.6–80.3	38.3–92.2	76.8–78.1	
Validated, *n* (%)	78 (100%)	40 (100%)	9 (100%)	27 (100%)	2 (100%)	
BMI						0.881 B)
Median (IQR)	25.8 (23.1–28.6)	25.7 (22.8–28.5)	26.0 (24.2–28.4)	26.0 (23.6–29.2)	26.4 (25.8–27.0)	
Range	18.3–35.2	18.3–34.5	18.5–32.8	18.6–35.2	25.2–27.5	
Validated, *n* (%)	77 (98.7%)	39 (97.5%)	9 (100%)	27 (100%)	2 (100%)	
Hospital						0.810 A)
Center 1	4 (5.1%)	2 (5.0%)	0	2 (7.4%)	0	
Center 2	56 (71.8%)	28 (70.0%)	8 (88.9%)	18 (66.7%)	2 (100%)	
Center 3	18 (23.1%)	10 (25.0%)	1 (11.1%)	7 (25.9%)	0	

A) Chi-squared test, B) Kruskal Wallis-test

*UICC* Union for international cancer control, *LN* Lymph node, *SLN* Sentinel lymph node, *ITC* Isolated tumour cell, *BMM* Bone marrow micrometastases, *CEA* Carcino embryonic antigen, *BMI* Body mass index, *IHC* immunohistochemistry

Overall, 28.2% of tumours were UICC stage I, while 71.8% were stage II. The median number of analysed LN was 23.5 (IQR 19.0–31.0). In 77 patients (98.7%), ≥ 12 LN were retrieved and analysed. In total, 12 patients (15.4%) received adjuvant chemotherapy because of present high risk factors [2] (Table 1).

For further analysis, the population was divided into four groups according to ± ITC and ± BMM status. Demographic information and tumour details of patients in the four groups are shown in Table 1. Overall, 51.3% were ITC− /BMM−, 11.5% of patients showed isolated ITC+ , while 34.6% patients showed BMM+. In two patients (2.5%) ITC+ /BMM+ were identified (Table 1).

### Kappa analysis

In Kappa analysis no association between the presence of ITC+ and BMM+ in stage I and II colon cancer patients was observed (kappa = − 0.13, 95%CI = − 0.4–0.14, *p* = 0.342).

### Multivariate Firth’s logistic regression analysis

In uni- and multivariable logistic regression analyses, the presence of BMM+ did not predict the occurrence of ITC in uni- and multivariable analyses (OR = 0.39, 95%CI: 0.07–1.50, *p* = 0.180 and OR = 0.74, 95%CI: 0.11–3.86, *p* = 0.730) (Table 2).

**Table 2:** Multivariate Firth’s logistic regression for prediction of BM and SLN

	Prediction of ITC+				Prediction of BMM+			
	Univariate analysis		Multivariate analysis		Univariate analysis		Mulitivariate analysis	
	OR (CI)	*p*-value A)	OR (CI)	*p*-value A)	OR (CI)	*p*-value A)	OR (CI)	*p*-value A)
*Isolated tumour cells*								
ITC −					Reference	0.180	Reference	0.519
ITC +					0.39 (0.07–1.50)		0.58 (0.09–2.95)	
*Bone marrow*								
BM −	Reference	0.180	Reference	0.730				
BM +	0.39 (0.07–1.50)		0.74 (0.11–3.86)					
*UICC stage*								
I	Reference	0.019	Reference	0.039	Reference	0.656	Reference	0.640
II	11.37 (1.37–1483.47)		10.27 (1.10–1727.27)		0.80 (0.30–2.19)		0.77 (0.25–2.31)	
*Lymphovascular invasion*								
Absent	Reference	0.007	Reference	0.030	Reference	0.515	Reference	0.555
Present	8.47 (1.84–40.32)		10.44 (1.24–151.56)		0.61 (0.11–2.59)		(0.07–3.53)	
*Grade*								
G2	Reference	0.945	Reference	0.075	Reference	0.211	Reference	0.135
G3	0.95 (0.22–3.47)		0.13 (0.01–1.20)		1.86 (0.70–4.99)		2.48 (0.75–8.87)	
*Tumour localisation*								
Right colon	Reference	0.332	Reference	0.283	Reference	0.020	Reference	0.023
Transverse colon	3.42 (0.63–18.89)		4.57 (0.45–69.42)		0.20 (0.03–0.83)		0.20 (0.03–0.87)	
Left colon	1.93 (0.47–8.99)		0.72 (0.10–4.58)		0.31 (0.11–0.83)		0.29 (0.09–0.85)	
*Hospital*								
Center 1	Reference	0.478	Reference	0.344	Reference	0.814	Reference	0.987
Center 2	2.03 (0.19–277.11)		0.96 (0.05–156.24)		0.56 (0.08–3.89)		1.15 (0.12–11.90)	
Center 3	0.77 (0.03–120.42)		0.22 (0.00–43.09)		0.65 (0.08–5.11)		1.21 (0.11–15.05)	
*Year of surgery*								
2001–2003	Reference	0.196	Reference	0.304	Reference	0.528	Reference	0.406
2004–2005	0.54 (0.09–2.64)		0.39 (0.05–2.35)		0.79 (0.27–2.27)		0.70 (0.21–2.22)	
2006–2007	2.23 (0.55–9.58)		1.75 (0.30–10.63)		1.53 (0.49–4.87)		1.71 (0.48–6.35)	

*OR* Odds ratio, *CI* Confidence interval, A) likelihood ratio tests, *UICC* Union for international cancer control, *ITC* Isolated tumour cell, *BMM* Bone marrow micrometastases

Conversely, the presence of ITC+ did not predict the occurrence of BMM+ (OR = 0.39, 95%CI: 0.07–1.50, *p* = 0.180 and OR = 0.58, 95%CI: 0.09–2.95, *p* = 0.519) (Table 2).

Higher UICC tumour stage (OR = 10.27, 95%CI:1.10–1727.27, *p* = 0.039) and present LVI (OR 10.44, 95%CI:1.24–151.56, *p* = 0.030) were independent predictors for the presence of ITC+ (Table 2).

Right-sided tumour localisation was predictive of BMM+ in univariate and multivariate analyses (compared to tumour localisation in the transverse colon: OR = 0.20, 95%CI:0.03–0.87 and tumour localisation in the left colon: OR 0.29, 95%CI:0.09–0.85, *p* = 0.023) (Table 2).

### Multivariate survival analysis

In univariate and multivariate analyses for OS, no significant association of any of the analysed parameters was found (Table 3).

**Table 3:** Multivariate survival analysis

	Overall survival				Disease-specific survival				Recurrence-free survival			
	Univariate analysis		Multivariate analysis		Univariate analysis		Multivariate analysis		Univariate analysis		Multivariate analysis	
	HR (CI)	*p*-value	HR (CI)	*p*-value	HR (CI)	*p*-value	HR (CI)	*p*-value	HR (CI)	*p*-value	HR (CI)	*p*-value
*ITC*												
Negative	Reference	0.401	Reference	0.208	Reference	0.277	Reference	0.197	Reference	0.581	Reference	0.601
posItive	1.63 (0.55–4.84)		2.85 (0.58–14.02)		1.78 (0.67–4.76)		2.72 (0.61–12.13)		1.57 (0.34–7.30)		2.04 (0.15–27.45)	
*BM*												
Negative	Reference	0.426	Reference	0.324	Reference	0.410	Reference	0.378	Reference	0.645	Reference	0.673
Positive	1.40 (0.61–3.21)		1.67 (0.61–4.58)		1.39 (0.64–3.05)		1.53 (0.60–3.89)		0.74 (0.20–2.78)		1.49 (0.24–9.22)	
*UICC stage*												
I	Reference	0.375	Reference	0.259	Reference	0.364	Reference	0.239	Reference	0.104	Reference	0.487
II	0.68 (0.29–1.58)		0.46 (0.12–1.81)		0.69 (0.31–1.52)		0.46 (0.13–1.71)		4.11 (0.52–32.19)		2.39 (0.18–31.19)	
*Lymphovascular invasion*												
Absent	Reference	0.443	Reference	0.948	Reference	0.233	Reference	0.976	Reference	0.327	Reference	0.803
Present	1.67 (0.49–5.68)		0.94 (0.15–5.78)		2.04 (0.69–6.01)		1.02 (0.21–5.07)		2.33 (0.49–11.02)		1.37 (0.12–15.84)	
*Grade*												
G2	Reference	0.279	Reference	0.315	Reference	0.171	Reference	0.153	Reference	0.108	Reference	0.056
G3	1.63 (0.69–3.88)		2.04 (0.52–8.02)		1.81 (0.80–4.12)		2.72 (0.71–10.40)		2.78 (0.83–9.30)		10.19 (0.82–127.14)	
*Tumour localisation*												
Right colon	Reference	0.400	Reference	0.534	Reference	0.190	Reference	0.369	Reference	0.019	Reference	0.231
Transverse colon	2.17 (0.71–6.64)		1.61 (0.39–6.60)		2.27 (0.74–6.97)		1.89 (0.46–7.77)		10.64 (1.10–102.49)		7.04 (0.37–134.53)	
Left colon	1.48 (0.58–3.76)		1.99 (0.54–7.30)		2.04 (0.85–4.94)		2.15 (0.66–7.03)		8.82 (1.08–71.77)		5.70 (0.44–73.52)	
*Chemo therapy*												
No	Reference	0.077	Reference	0.158	Reference	0.659	Reference	0.908	Reference	0.278	Reference	0.297
Yes	0.24 (0.03–1.76)		0.26 (0.03–2.25)		0.77 (0.23–2.57)		1.09 (0.27–4.37)		2.20 (0.58–8.40)		3.26 (0.36–29.41)	
*Sex*												
Male	Reference	0.156	Reference	0.687	Reference	0.116	Reference	0.726	Reference	0.277	Reference	0.747
Female	0.55 (0.24–1.27)		0.80 (0.28–2.33)		0.53 (0.24–1.18)		0.84 (0.32–2.20)		0.51 (0.15–1.75)		0.75 (0.13–4.28)	
*Age*												
< 70	Reference	0.131	Reference	0.231	Reference	0.162	Reference	0.077	Reference	0.595	Reference	0.381
≥ 70	2.06 (0.76–5.55)		2.03 (0.62–6.69)		1.86 (0.75–4.64)		2.68 (0.86–8.34)		1.42 (0.38–5.36)		2.83 (0.26–30.18)	
*BMI*												
< 25	Reference	0.288	Reference	0.259	Reference	0.365	Reference	0.154	Reference	0.146	Reference	0.163
≥ 25	0.64 (0.28–1.46)		0.59 (0.24–1.48)		0.70 (0.32–1.51)		0.52 (0.21–1.28)		0.41 (0.12–1.41)		0.30 (0.05–1.69)	

*HR* Hazard ratio, *CI* Confidence interval, A) Likelihood ratio tests, *UICC* Union for international cancer control, *ITC* Isolated tumour cell, *BM* Bone marrow, *BMI* body mass index

### Univariate 5-year survival rates

Five-year survival rate regarding OS was 85.0 (74.6–96.8) for ITC– /BMM–, 76.2 (52.1–100.0) for ITC+ /BMM–, 77.8 (63.6–95.2) for ITC– /BMM+ and 0.0 for ITC+ /BMM+.

Five-year DSS was 85.0 (74.6–96.8) for ITC– /BMM–, 63.5 (37.7–100.0) for ITC+ /BMM–, 74.1 (59.3–92.6) for ITC– /BMM+ and 0.0 for ITC+ /BMM+.

Five-year RFS was 89.5 (80.2–99.8) for ITC– /BMM–, 71.4 (44.7–100.0) for ITC+ /BMM–, 88.3 (76.7–100.0) for ITC– /BMM+ and 0.0 for ITC+ /BMM+.

For ITC+ patients (*n* = 11) and ITC– patients (*n* = 67) the OS, DSS and RFS where 62.3 (38.9–99.9) vs. 82.1 (73.4–91.8) (*p* = 0.401), 51.9 (28.7–93.9) vs. 80.6 (71.7–90.6) (*p* = 0.277) and 71.4 (44.7–100.0) vs. 89.0 (81.6–97.0) (*p* = 0.581).

For BMM+ patients (*n* = 29) and BMM– patients (*n* = 49) OS, DSS and RFS where 72.4 (57.9–90.7) vs. 83.6 (73.9–94.7) (*p* = 0.426), 69.0 (54.0–88.0) vs. 81.5 (71.3–93.2) (*p* = 0.410), 88.6 (77.2–100.0) versus 86.9 (77.7–97.3) (*p* = 0.645).

### Adjusted survival curves

**Fig. 2:**
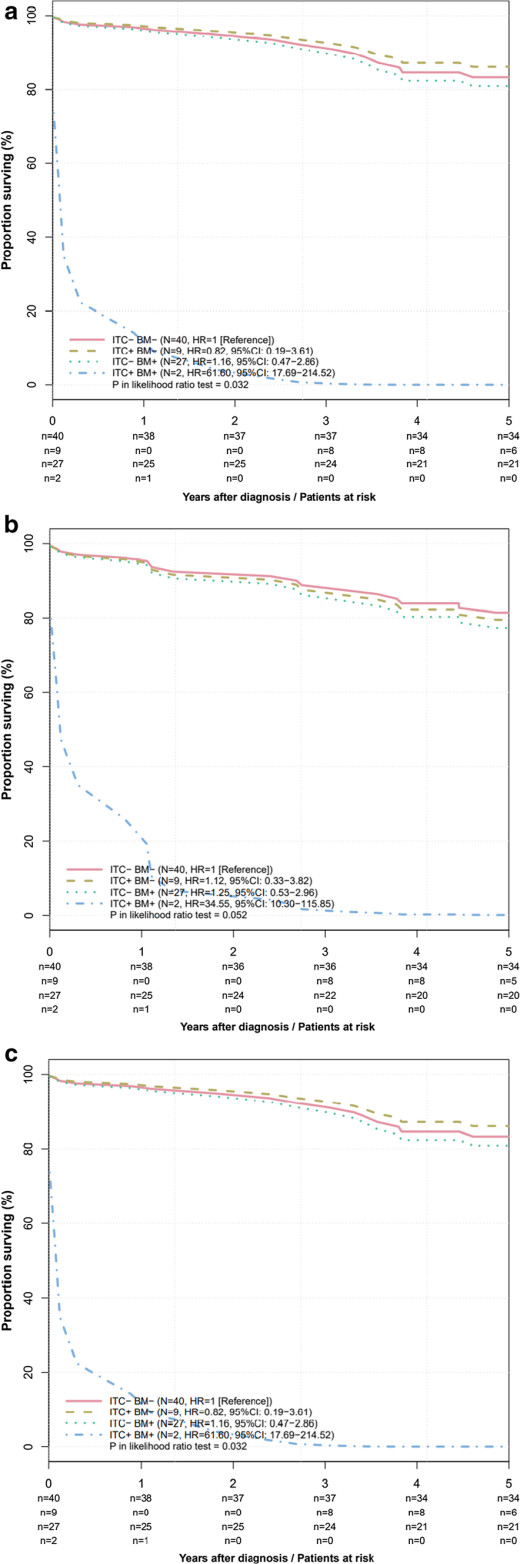
Adjusted survival curves for overall survival (**a**), disease-specific survival (**b**) and recurrence-free survival (**c**). Stratified for stage, center, using sandwich estimator for variances

Analysing ITC and BMM as one single factor (ITC– /BMM–, ITC+ /BMM–, ITC– /BMM+, ITC+ /BMM+) instead of fitting them as two independent factors (Table 3) reveals the impact of the simultaneous occurrence of ITC+ and BMM+ despite the low number of patients in this subgroup (*n* = 2). For OS and RFS, a distinctly worse survival is seen for patients with ITC+ /BMM+ compared to the three other groups (HR = 61.6; 95%CI:17.69–214.52; *p* = 0.032) (Fig. 2a) and (HR = 61.60; 95%CI:17.69–214.52; *p* = 0.032) (Fig. 2c). For DSS, no significant difference was observed between the four subgroups (HR = 34.55; 95%CI:10.30–115.85; *p* = 0.052) (Fig. 2b).

## Discussion

The objective of the present study was to investigate the interdependent relationship and respective prognostic relevance of early tumour cell presence in the first draining LN (SLN) and the BM in patients with stage I and II colon cancer, thereby gaining insight into the first steps of tumour cell dissemination. The prevailing doctrine currently is that tumour cell spread occurs in a sequential order, first via LN and later systemically to distant sites. One of the first descriptions of this process was made almost two decades ago in patients with melanoma (incubator hypothesis) [24]. Nevertheless, this study shows that the appearance of ITC in SLN and BMM occurred independent from each other in patients with stage I and II colon cancer, suggesting an early, simultaneous and non-interdependent spread to the LN and the BM. Neither ITC in LN nor BMM are thereby recognised risk factors for patients with colon cancer. Still, we were able to show that patients with a simultaneous spread to both these sites had a significant worse OS and RFS.

According to the current tumour classification systems, patients with micrometastases in LN are considered node-positive while patients with ITC are considered node-negative [7–9]. As nodal status distinguishes stage I and II from stage III and therefore divides patients not automatically qualifying for adjuvant chemotherapy from patients routinely receiving chemotherapy, LN status has a huge impact on further oncologic treatment. The distinction of micrometastases and ITC had been introduced in the 6th edition of the TNM classification [7, 40]. However, evidence-based data supporting a cut-off at 0.2 mm are not available. In the meantime, the prognostic importance of ITC has been repeatedly demonstrated, challenging their attribution to node-negativity [11, 12, 15, 16, 41].

ITC detected in SLN might thereby represent the very earliest step of lymphatic tumour spread as SLN have been shown to represent the first draining LN in the hierarchical lymphatic draining system of colon cancer and harbour metastases significantly more often than all other LN [6, 14–17]. Mapping and in-depth analysis by immunohistochemical stainings of multi-level sections of SLN provides therefore a more accurate staging and triggers through stage migration (Will Rogers phenomenon) more homogenous groups that all show an improved outcome [42].

Analysis of BM in colon cancer patients is not routinely performed. This despite existing evidence that BMM are associated with worse DFS and OS in stage I–III colon cancer [30, 32, 43]. The reports focus on stage I–III colon cancer, leaving the possibility that stage III with macro-metastatic tumour spread to LN has influenced the results by representing an already established and advanced tumour spreading state. Narrowing the analysed population to only stage I and II reveals a more homogenous group possibly representing a similar biological state.

In accordance with the common understanding of tumour spread in colon cancer, stage (UICC stage I vs II) and LVI were predicting factors for ITC+ while BMM+ was only indicated by the tumour site and in particular not by stage or LVI. The correlation of LVI and early tumour spread, i.e. ITC+, supports current practice of assigning those patients to systemic adjuvant therapy [2]. The fact that right sided tumours were associated with BMM+ might help explain why those tumours are generally suspected to have a worse prognosis [44], although we have not observed in our population a difference in outcome in dependence of the tumour localisation.

ITC+ and BMM+ indicate two distinct ways of tumour spread which nevertheless occurred simultaneously even at this early stage. Therefore, the haematologic and prognostic relevant tumour spread in stage I and II colon cancer challenges the general tenet of metastatic tumour spread [5, 45]. Basic science and advances in molecular biology will certainly deepen our yet basic understanding of biology and tumour spread [21, 22, 25, 26, 46, 47]. Furthermore, genetic analysis could facilitate the determination of the origin of BMM and provide additional evidence in support of the theory of early metastatic spread.

Our study has limitations we want to acknowledge. On the one hand, this it is a cohort study and not a randomized clinical trial, so there may be confounding factors that we could not control for. However, patient groups are comparable in terms of baseline characteristics. Second, this analysis is a subset of a larger population, and in some groups only a few patients remained. In particular we underline that only two patients presented with ITC+ /BMM+. It might be that ITC+ /BMM+ combination represents a subgroup that is not seen that often. But as these patients show a distinct worse outcome compared to isolated ITC+ or BMM+ alone, or to no tumour cell spread, further research in the area is needed and should bring additional evidence.

This is, to our knowledge, the first study investigating risk factors for a worse prognosis in node-negative colon cancer patients who underwent SLN mapping and BM analysis. We have thereby been able to find evidence that the simultaneous presence of ITC in SLN and BMM  might represent a significant risk factor for a decreased OS and RFS and certainly warrants further investigation.

## Conclusions

Our results indicate that the simultaneous and not interdependent presence of ITC in SLN and tumour cells in the BM may be considered as a risk factor for patients with non-metastatic colon cancer.

Our findings suggest that relevant lymphatic and haematologic tumour spread occurs already at such early stages and significantly impairs prognosis, thereby challenging the prevalent theory of sequential tumour progression and proposing the possible need to reconsider the common assumptions on tumour spread. The results further underline the importance of in-depth analysis and assessment of additional risk factors in colon cancer patients like ITC in SLN and tumour cells in the BM.
